# Supplementary figures and images for: Left-right cortical interactions drive intracellular pattern formation in the ciliate Tetrahymena
Source: PLoS Genet. 2025 Jun 2;21(6):e1011735. doi: 10.1371/journal.pgen.1011735 (PMC12151471; doi:10.1371/journal.pgen.1011735)

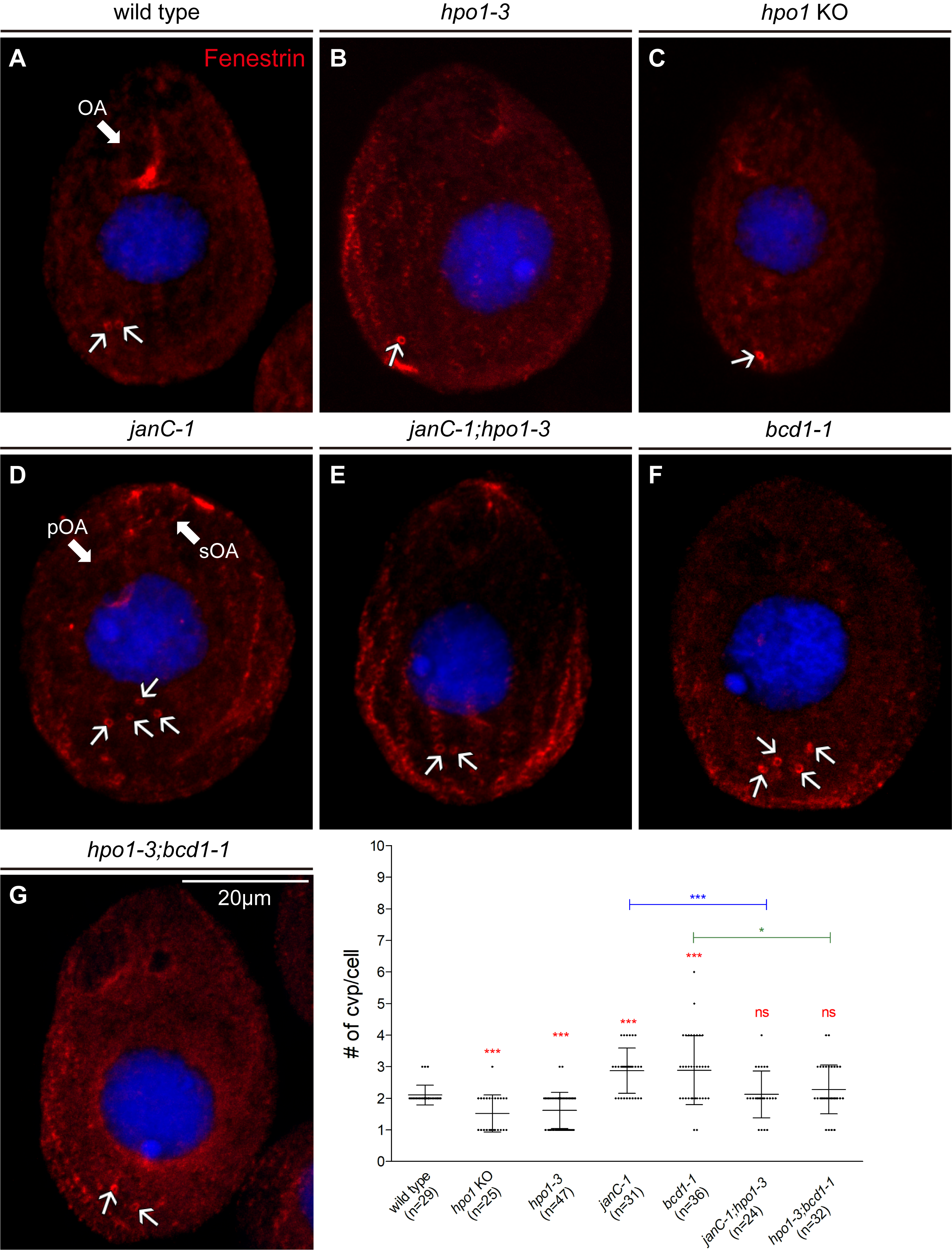

Supplement: S1 Fig — All genotypes are homozygous as indicated in the figure panels. (A-G) Confocal images of wild type (A), single mutants (B-D, F), and double mutants (E, G). The cells were labeled with the anti-fenestrin antibody (red), and DAPI (blue) after incubation for 4 hours at 38°C. The white arrows point to the CVPs. (H) The graph quantifies the number of CVPs per cell. The bars represent the mean ± SD. The number of cells scored is displayed in each of the bar. A two-tailed unpaired t-test was executed for statistical significance. ns: not significant. Stars indicate statistically significant (*: P < 0.05, **: P < 0.01, and ***: P < 0.001, ns: not significant). Red stars indicate statistically significant differences as compared to the wild-type. Abbreviations: pOA, primary OA; sOA, secondary OA (in the janC-1 background). (TIF) [file pgen.1011735.s001.tif]

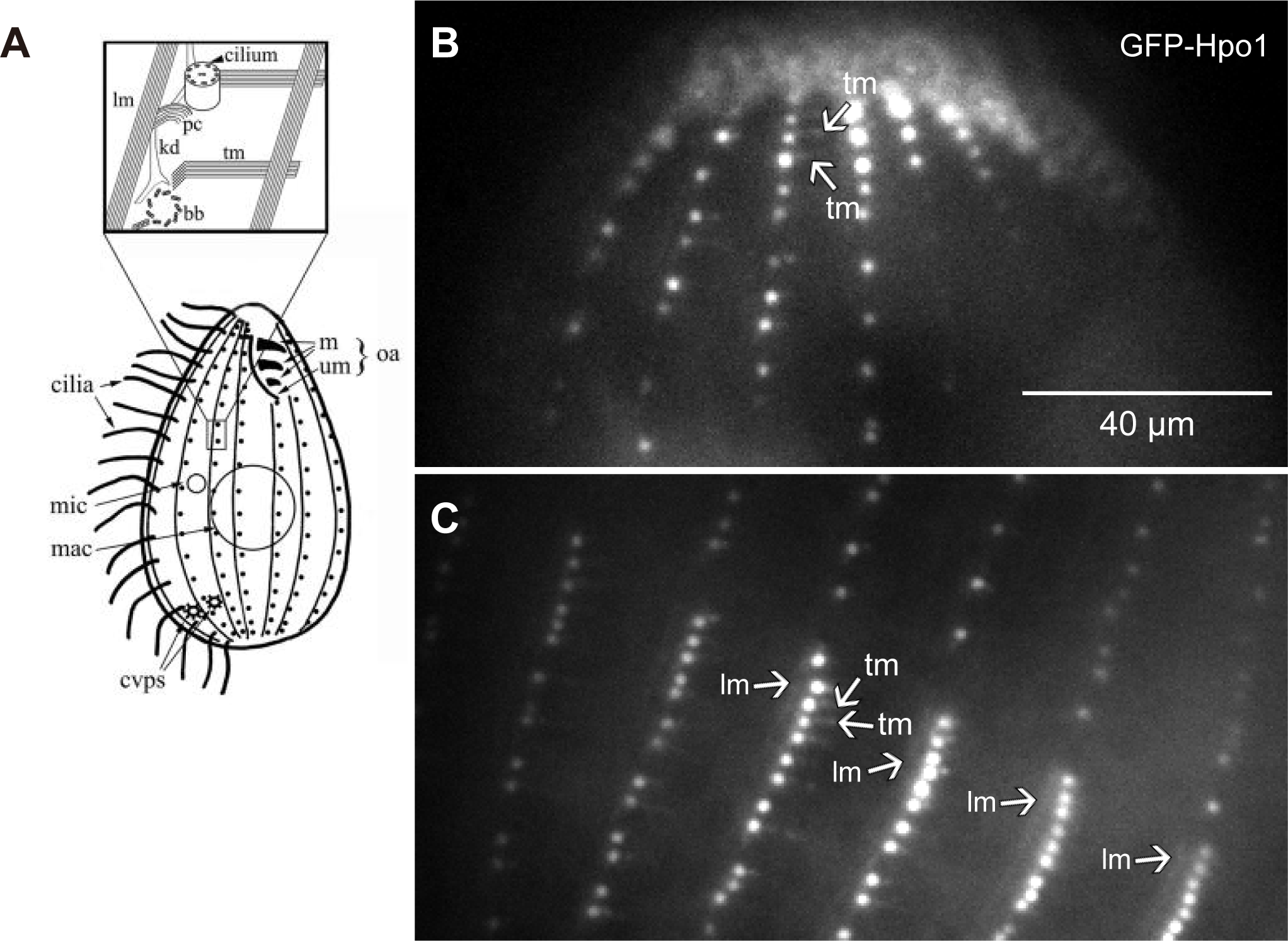

Supplement: S2 Fig — (A) An illustration of T. thermophila with the magnified inset describing a portion of the cell cortex (reproduced with the publisher’s permission from Fig 1 [67]. (B, C) Still images of live cells expressing GFP-Hpo1 that localizes at positions consistent with the basal bodies and microtubule bundles (indicated by arrows). Abbreviations: tm, transverse microtubule bundle; lm, longitudinal microtubule bundle; pc, postciliary microtubule bundle; bb, basal body; kd, kinetodesmal fiber (non-microtubular); m, membranelle M row; um, undulating membrane row; oa, oral apparatus; mic, micronucleus; mac, macronucleus; cvps, contractile vacuole pores. (TIF) [file pgen.1011735.s002.tif]

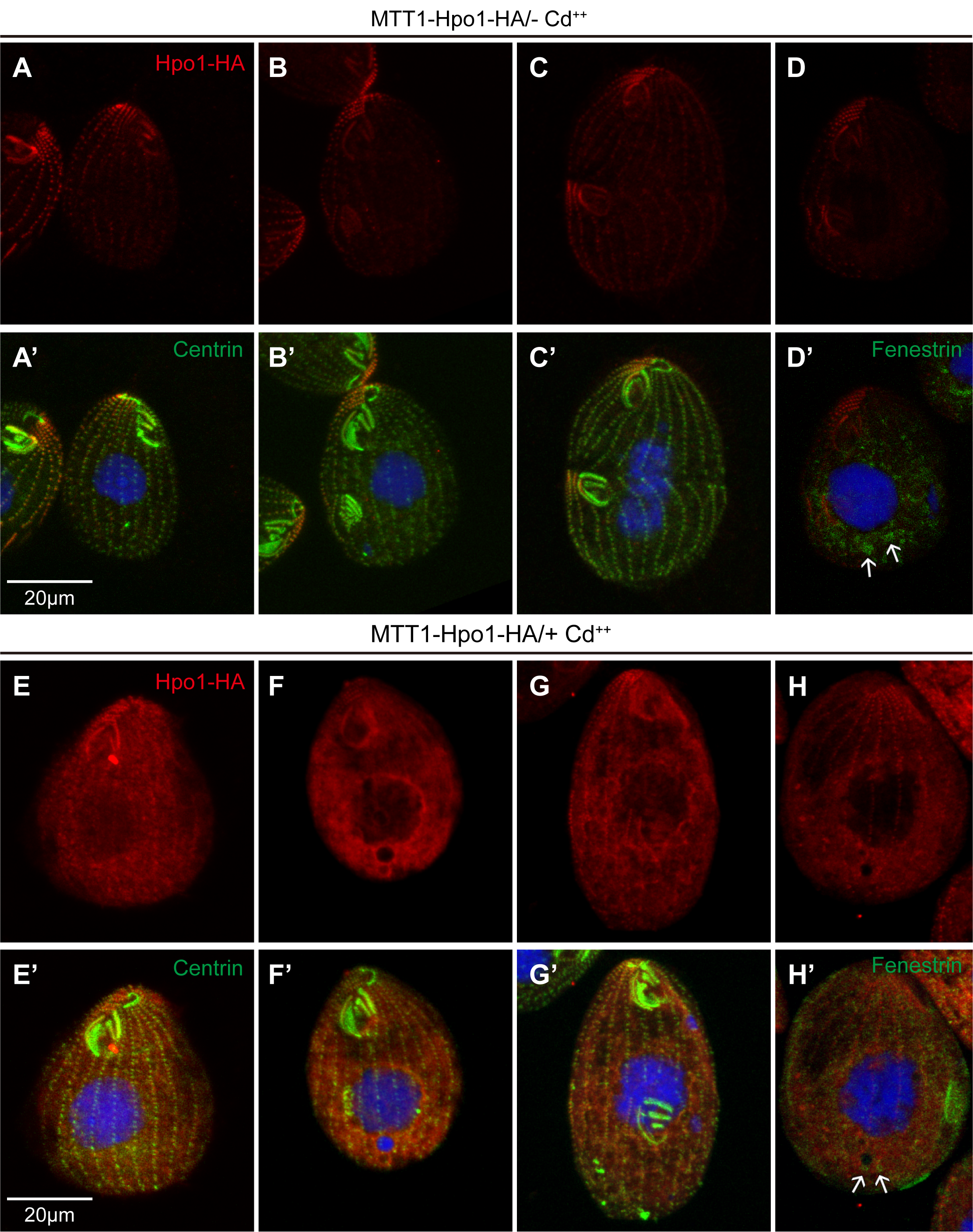

Supplement: S3 Fig — Cells carrying a transgene expressing Hpo1-HA under the MTT1 promoter were grown either without (A-D’) or with addition of 2.5 μg/ml cadmium chloride for 6 hr (E-H’), fixed and labeled with the anti-HA (red) and either anti-centrin (A’-C’; E’-G’) or anti-fenestrin (D’, H’) (green) antibodies and DAPI (blue). Note an accumulation of Hpo1-HA in the cell body of overproducing cells. Despite overproduction, the right-side and anterior gradients of Hpo1 are still apparent and the positions and number of OPs (E-G’ compare to A-C’) and the number of CVPs (H,H’ compare to D,D’) are unaffected. (TIF) [file pgen.1011735.s003.tif]

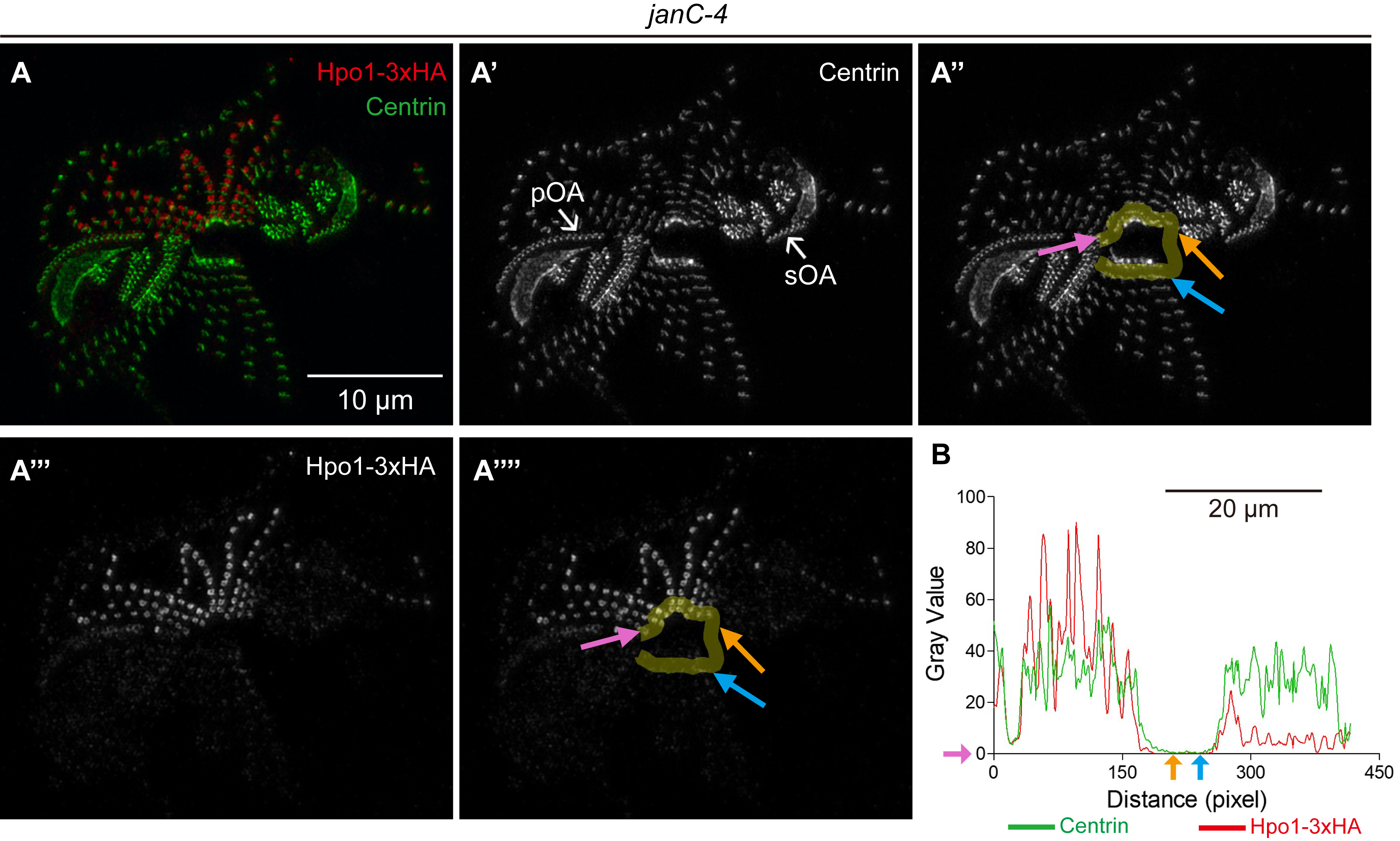

Supplement: S4 Fig — (A-A’”’) An SR-SIM image of Hpo1-3xHA and centrin in an apical cell fragment of the janC-4 homozygote. (A) The cell fragment was labeled with anti-HA (red), 20H5 anti-centrin antibody (green), and DAPI (blue) after overnight incubation at 30°C. Duplicates of single channel grey scale images for centrin (A’,A”) and Hpo1-3xHA (A”’, A’”’) are shown. The yellow lines mark the area used for measurements of signal intensity. The pink arrows show the start locations for signal intensity measurements. The orange and cyan arrow indicates initial and end point of gap, respectively. (B) The graph shows that signal intensity plots for Hpo1-3xHA (red line) and centrin (green line). (TIF) [file pgen.1011735.s004.tif]

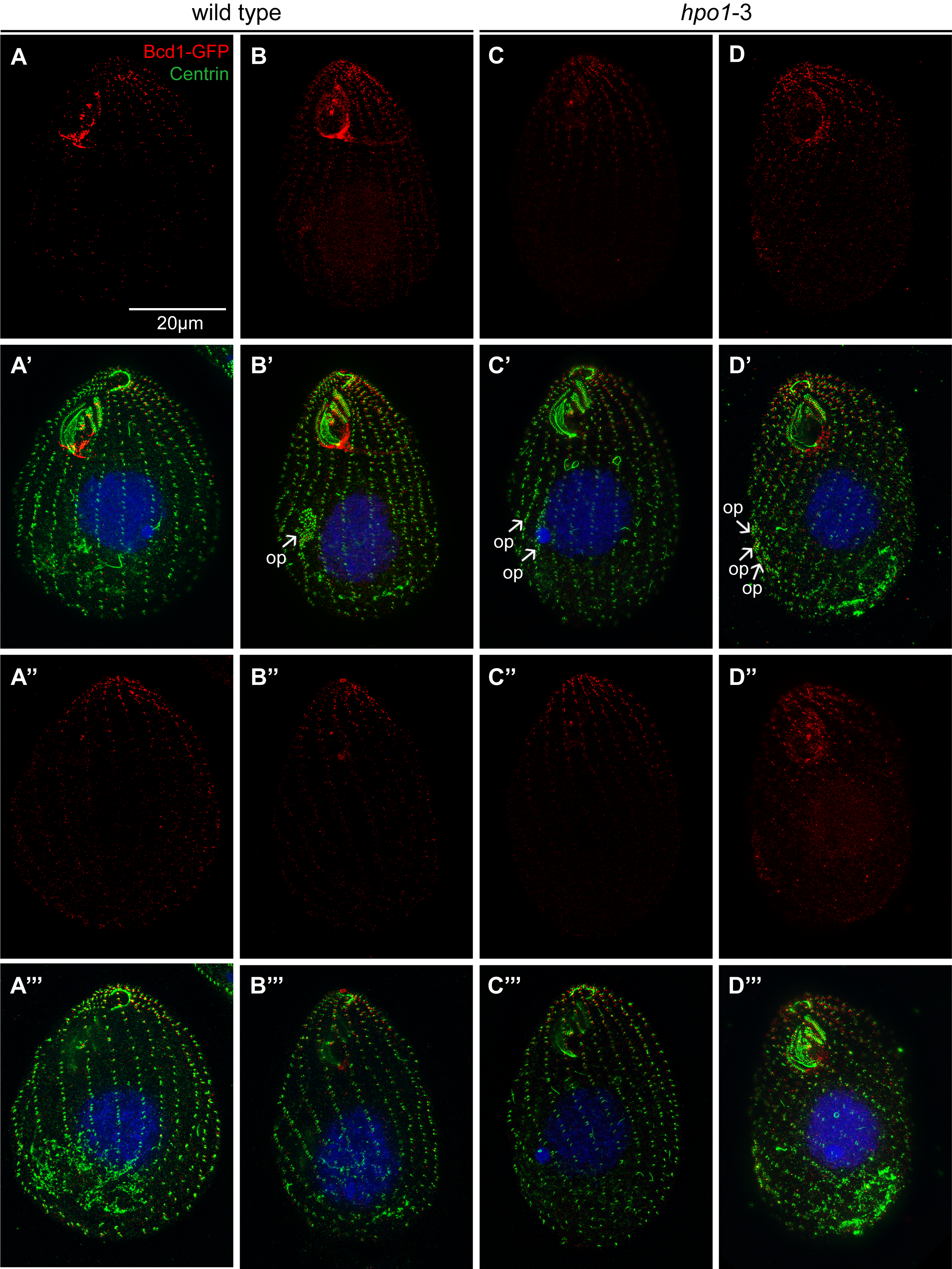

Supplement: S5 Fig — (A-D”’) Pairs of SR-SIM images showing two sides of the same cells that express Bcd1-GFP and are either otherwise wild-type (A-B”’) or hpo1-3 (C-D”’). The cells were labeled with the anti-GFP antibodies (red), 20H5 anti-centrin antibody (green), and DAPI (blue) after a period of growth for 4 hours at 39°C. (TIF) [file pgen.1011735.s005.tif]

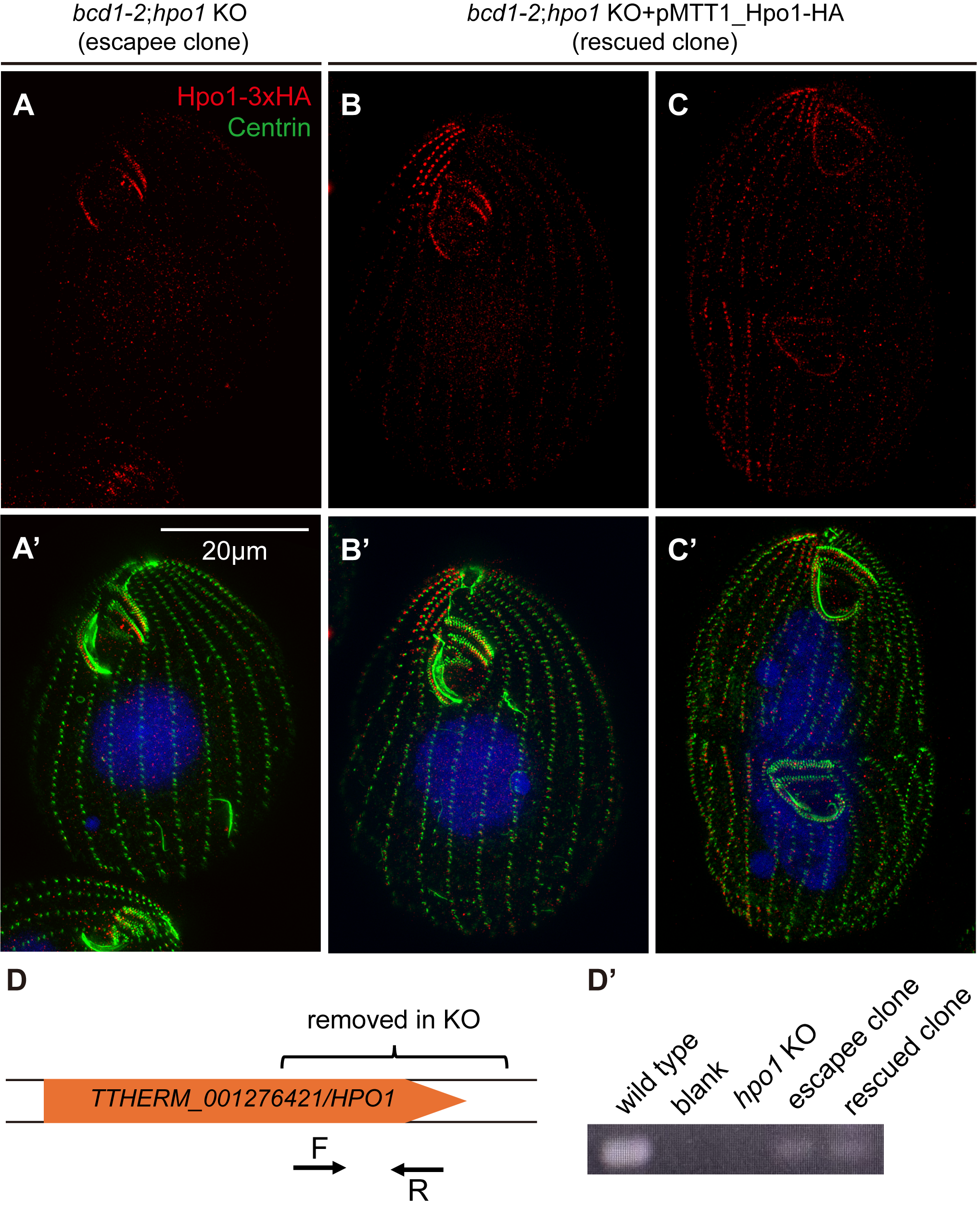

Supplement: S6 Fig — (A-C’) SR-SIM images clones selected from the population of mating heterokaryons homozygous in the micronucleus for bcd1-2 and hpo1-KO alleles that were either subjected to a mock biolistic bombardment (without transgene DNA) (A,A’) or biolistically transformed with a transgene encoding MTT1-Hpo1-3xHA (B-C’). The cells were labeled with anti-HA (red), 20H5 anti-centrin antibody (green), and DAPI (blue) after overnight incubation at 30°C. (D,D’) The diagram shows the positions of PCR primers designed for amplification of the portion of HPO1 gene sequence deleted in the hpo1-KO allele (D) and the gel image showing the PCR products amplified from the genomic DNA isolated either from the escapee clone or from rescued clone (D’). (TIF) [file pgen.1011735.s006.tif]
