# Supplementary material for: Left-right cortical interactions drive intracellular pattern formation in the ciliate Tetrahymena
Source: PLoS Genet. 2025 Jun 2;21(6):e1011735. doi: 10.1371/journal.pgen.1011735 (PMC12151471; doi:10.1371/journal.pgen.1011735)
Supplement: Attachment — Submitted filename: Response_to_Reviewers.docx [file pgen.1011735.s009.docx]

**Authors response to the reviewers’ comments:**

*We are grateful to the reviewers for their insightful evaluations. We have attempted to address every issue raised. The responses to the reviewers’ comments and the steps taken are described below, point-by-point:*

Reviewer #1: In the study entitled “Left-right cortical interactions drive intracellular pattern formation in the ciliate Tetrahymena”, Chinkyu Lee, Ewa Joachimiak, Wolfgang Maier, Yu-Yang Jiang, Karl F. Lechtreck, Eric S. Cole, and Jacek Gaertig identified, for the first time, the Tetrahymena gene HPO1 (TTHERM_001276421), which encodes a ARMC9-like protein responsible for the mutant phenotypes previously linked to Tetrahymena hypoangular 1 (hpo1) alleles. Homozygous hpo1 mutants exhibit multiple oral apparatus (OA) with abnormal positioning to the left or right of the normal site in the cell.
The authors provide strong evidence that hpo1 gene is responsible for these phenotypes by generating Tetrahymena strains carrying previously described hpo1 mutations and hpo1 knockout strains, both displaying similar phenotypes. They further localized the Hpo1 protein to ciliary basal bodies using a strain expressing Hpo1-GFP, or Hpo1-3xHA and assessed its overexpression phenotype. Their findings reveal that and Hpo1 protein preferentially localizes to anterior basal bodies along a subset of ciliary rows on the cell’s right side, with levels highest at the most anterior basal bodies and decreasing along the row length. These gradients are crucial for defining contractile vacuole pores positioning and ensuring OA exclusion from the right side of the cell.
Additionally, the authors explored genetic interactions between the genes bcd1 (cell’s left side-enriched factor) and janA (encodes a Polo kinase), which localizes to the left-to-dorsal circumferential region of Tetrahymena. They found that Hpo1 interacts with Bcd1, and the simultaneous loss of both proteins is lethal, severely disrupting OA positioning and organization. This interesting study builds on the authors' previous work on the gradient-forming factors involved in ciliate morphogenesis, as well as the maintenance and organization of complex cortical organelles during cell division.
The paper is worthy of publication; however, incorporating some minor suggestions will improve the manuscript.
• Figure 5 – In both confocal and SR-SIM images, it is challenging to observe the increased number of rows with enriched Hpo1-3xHA in the janC-4 background compared to the wild type, as shown in panel E. Therefore, these images may not be the most representative. For the confocal images, it would be helpful to include a wild type cell for comparison.

*The difference in the number of rows between the wild type and the janC-4 mutant is small (graphs in Figure 5E,F). However, the janC-1 cell shown in panel 5D-D’ has 9 strong Hpo1-3xHA rows, which is in the upper range for the janC-1 mutant. While not perfect, the figure conveys the following observations: 1) both the primary and secondary OA are located at the boundaries of the strong Hpo1 sector on the cell’s right side, 2) besides a small expansion of the Hpo1 sector (which could be a scaling response to the increased total number of rows), its pattern is not grossly altered in the janC-4 background.*

• Figure 7 – In panels (C-C’’), it would be helpful to include an SR-SIM image of a wild type cell’s apical region for comparison with the images of a cell expressing Hpo1-3xHA (red) in the bcd1-2background.

*The wild-type top view image of centrin/Hpo1-3xHA along with the signal intensity plot is a part of Figure 4D-E. Unfortunately, images in which the cell fragment is properly positioned and well preserved are rare. While we did not include a control top view image in Figure 7, we revised the corresponding part of Results and the legend of Figure 7 to refer the reader to the wild-type image in Figure 4D for comparison.*

• A schematic similar to Figure 6K would be beneficial to summarize the findings from Figures 7 and 8, particularly regarding the genetic interaction between *bcd1-2* and *hpo1-3*.

*We made a few changes to help the reader navigate this part of Results and help to more easily compare the distribution of Hpo1 between the wild-type and bcd1-2 backgrounds. There are three relatively subtle effects of bcd1-2 expression on the distribution of Hpo1: 1) flattening of the AP gradient, 2) shift in the position of the “contrast row” correlated with the shift in the OP positions caused by expression of bcd1-2 and 3) ambiguity in the definition of the contrast row on the ventral surface in the interphase cells (before the OP formation).*

*Regarding the effect 1), in figure 7, we rearranged the positions of the signal intensity plots so that they are next to the source images used for tracing.*

*Regarding the effects 2) and 3), we revised figure 8. The original figure 8 contained only images of Hpo1 in the bcd1-2 mutant background (a more complete set of images using those in the wild-type background were included in the original figure S6). In the revised figure 8, we included images of Hpo1-3xHA in both the wild-type and bcd1-2 background so that the reader can compare them side-by-side. We deleted the original figure S6, accordingly.*

• It may be possible to combine Figures 7 and 8 if the confocal images from Figure 8 are moved to the supplementary figures. However, it is essential to clearly indicate in the panels that the cells in Figure 8 are homozygous for bcd1-2.

*We have not done it, but as mentioned above, we made changes in both figure 7 and 8 to improve their design and make it easier to compare the different genotypes. As requested, in the revised figure 8, we included the genotype labels.*

• Figure 9 – It would be helpful to indicate in the panels that the cells in the images are bcd1-2;hpo1-KO double homozygotes.

*We added the genotype labels to panels in figure 9 as suggested.*

• Figure S4 – Although this information is provided in the legend, it would be helpful to indicate in the figure itself that the panels show janC-4 homozygotes.

*We added the genotype labels to figure S4 as suggested.*

• Figure S5 – As the authors mention, the Bcd1-GFP signal is indeed quite weak. However, it appears to be even weaker in hpo1-3 (panels C-D’’’). Is this observation accurate?

*To our eyes, there is no obvious effect of hpo1-3 expression on the pattern of Bcd1-GFP, but the detection of Bcd1-GFP was challenging (likely due to its low levels of expression), and the question of whether the Hpo1 deficiency affects the pattern of Bcd1 may need to be studied further with a more sensitive imaging approach. Note however, that a negative result (that Hpo1 does not regulate the distribution of Bcd1) could be correct; Hpo1 may regulate the activity of Bcd1 but not its distribution.*

Reviewer #2: Summary:
In this manuscript entitled “Left-right cortical interactions drive intracellular pattern formation in the ciliate Tetrahymena” (PGENETICS-D-25-00071), the authors report the identification of the protein encoded by the gene mutated in hypoangular 1 mutants of Tetrahymena as a ARMC9-like protein. These mutants have defects in circumferential patterning detected by shortening of the distance between the oral apparatus and the contractile vacuole pores. This is caused by a progressively rightward shift in the formation of a new oral appartus each cell division.By using whole-genome sequencing and variant analysis of wild-type and mutant genetic segregants, the authors were able to convincing identify the HPO1 gene. The original mutant alleles appeared to be hypomorphs and a gene knockout caused a more penetrant/severe phenotype. The hpo1 protein was shown to localize to the cells right lateral side. The shift of the oral primordium in that direction suggests that normally, the hpo1 gene product acts negatively to restrict the formation of a new oral apparatus where hpo1 is present. The hpo mutants not only shift the position of oral apparatus, but exhibit multiple foci of oral basal body poliferation, similar to the bcd1 mutants. The authors show that the bcd1/hpo1 double mutant cells result in cell lethality. This synthetic lethal phenotype is significant as Bcd1 is localized to the region to the left of the oral primorium. The authors propose that the two gene products act synergistically to prevent new oral apparatus formation to the left and the right of the row topped by the old oral apparatus. This study provides one of the first identifications of gene that controls left-right asymmetry in Tetrahymena.

Strengths:
The study is carefully executed and the experiments well described. The identification of the hpo1 protein is one of the first descriptions of the action of a protein that controls circumferential patterning in these highly structured unicellular eukaryotes, Tetrahymena. The genetic interactions and detailed cellular localization of the hpo1 protein in both Janus A (JanA) and broaden cortical domains (Bcd1) proteins provides important insight into the interplay into these major patterning determinants. This study reveals that multiple proteins with specific localizations provides the groundwork to future studies to characterize the precise signaling events needed for a Tetrahymena cell to precisely produce a daughter cell each cell division.

Weaknesses/comments to address:
1.There is little discussion as to how this ARMC9 protein may act to repress oral formation. Given that ARMC9 proteins are found in basal bodies and cilia in mammalian cells, the authors postulate that its role in circumferential patterning may be neo-functionalization. As a genetic definition, this is sufficient, but we don’t really learn of any possible molecular action through their findings or discussion.

*The function of canonical ARMC9 has started to emerge only recently. Coincidently, our laboratory was first to report that ARMC9 localizes to the distal tip of cilia and regulates the size of the ciliary distal segment in Tetrahymena (only recently confirmed by Wallingford group in Xenopus). In mammalian cells, ARMC9 is enriched at the basal body of the primary cilium and moves to the ciliary tip upon activation of hedgehog signaling. At the molecular level, ARMC9 forms a complex with other proteins that regulate microtubule assembly/disassembly. Within this complex, ARMC9 may function as a scaffold for several other ciliary proteins (Saunders et al nature Structural Biology 2025). The interaction partners of Hpo1 are unknown and the only connection between Hpo1 and ARMC9 is the localization to the ciliary basal body.* *We revised the Discussion by adding a paragraph that informs about the molecular function of the canonical ARMC9 and speculates about the function of Hpo1: “ARMC9 acts as a scaffold to form a complex with additional conserved ciliary tip-enriched proteins that plugs the plus end of microtubules and attenuates the assembly rate (Saunders et al 2025). Besides sharing the domain organization and weak homology, the only share property of Hpo1 and ARMC9 is the localization to the BBs. We speculate that Hpo1 acts as a scaffold for proteins that inhibit the proliferation of BBs destined to form the OP, possibly by inhibiting the growth of plus ends of triplet microtubules.”.*

2. Page 12. The heading states that Hpo1 interacts with Bcd1. As the results reveal a synthetic lethality/genetic interaction, I would suggest making the heading reflect the data. When this reviewer reads X interacts with Y, I expect of see some protein-protein interaction. Please provide a more specific heading.

*We revised the heading as follows: “Hpo1 interacts genetically with Bcd1, a left side-enriched OP positioning factor”.*

3. Page 19. In the discussion of the JanC/Hpo1 double mutant phenotype, i.e. the primary and secondary oral apparati are closer, the authors indicate that suggest that the hpo1 gene product likely restricts the position of oral appartus formation of both the primary and secondary oral apparati. From the images shown in figure 6, it was not clear whether both the position of primary and secondary oral apparati are shifted equally.

*We rewrote this section. It is straightforward to distinguish between the primary and secondary OA in the single janus mutant, because the secondary OA is invariably underdeveloped, and often inverted in its internal polarity. This distinction is less obvious and often not apparent in the double mutant janC-1;hpo1, likely because the two OAs, as they move toward each other, become similar, suggesting that the secondary OA normalizes as it moves closer to the ventral surface occupied by the primary OA. Several images of dividing cells “caught” in the course of the ”slippage” in which the parental OAs are still separated but the OPs are immediately adjacent, suggest that the directions of slippage for the primary and secondary OA are frequently opposite, which result in a collision of the two OPs as shown in the drawing in Figure 6K. In this drawing, the two OPs move into the right side sector normally occupied by Hpo1. We cannot however exclude a possibility that the directions of slippages of the primary and secondary OAs are opposite, that is that the primary OA moves to the left and the secondary OA moves to the right. In this model, the function of the Hpo1 sector would be to anchor the OPs at the margins of the Hpo1 domain (rather than to repel it from the right sector). We believe that this manuscript clearly shows that both Hpo1 sector margins are associated with oral development and that Hpo1 keeps the two OAs at the distance. Further work with additional markers of the ventral and dorsal surface (which are not currently available), could clarify the directions of slippage. We revised the relevant part of Results to be more accurate.*

4. It would be interesting to discuss why the Hpo1 and Bcd1 show synthetic lethality but not the JanC and Hpo1.

*This is an excellent question that prompted us to revise relevant portions of the Results and Discussion sections. We can speculate that janus mutations have a smaller impact on the cortical pattern as compared with bcd1-1. Janus mutations cause an appearance of a single incomplete OA on the dorsal side and a mild expansion of the CVP sector. In contrast, both hpo1-KO and bcd1-1 confer multiple OPs, and extensive lateral shifts in the OP positions. Furthermore, the phenotype of double mutants hpo1-KO;bcd1-2 revealed that either Hpo1 or Bcd1 are required to generate cues for controlling the position, size and internal organization of the OA. The actual reason why the double mutants stop multiplying is likely a cell cycle arrest. There is extensive literature about the cortical-nuclear interactions in ciliates. The cortex strongly influences the nuclei, for example by determining their AP and C positions within the endoplasm during cell division (we now cite multiple relevant papers). On the other side, a mutation in the micronuclear telomere sequence causes a cell cycle arrest and disturbs oral development (Kirk and Cole, EC, 2008). Thus the cortical-nuclear interactions are reciprocal. The terminal phenotype of the cell-cycle arrested double (hpo1-Ko;bcd1-2) mutants resembles that of the psm (pseudomacrostomal) mutants in which the cell cycle arrest is associated with an enlarged and grossly misshaped OA (Frankel et al 1984). We can therefore speculate the cell cycle arrest in the hpo1-Ko;bcd1-2 double mutants is secondary to the gross disorganization of the oral apparatus and that the cell cycle arrest could be a result of activation of an unknown checkpoint that monitors the integrity of oral structures. We have revised portions of the Discussion to bring more clarity to the nature of the terminal double mutant phenotype.*

Minor comments:
The second paragraph at the top of page 14 is one sentence and appears to be a statement without context. Was this an error during editing? What this suppose to be the end to the previous paragraph.

*We revised this portion of the manuscript to improve the logical flow.*
